# LINC00319-Mediated miR-3127 Repression Enhances Bladder Cancer Progression Through Upregulation of RAP2A

**DOI:** 10.3389/fgene.2020.00180

**Published:** 2020-03-03

**Authors:** Xiaoqing Wang, Ran Meng, Qing-Mei Hu

**Affiliations:** ^1^Department of Operation Room, Shangqiu First People’s Hospital of Henan, Shangqiu, China; ^2^Department of Urology, Shangqiu First People’s Hospital of Henan, Shangqiu, China

**Keywords:** miR-3127, RAP2A, LINC00319, bladder cancer, lncRNA

## Abstract

Recent studies suggested that microRNA-3127 (miR-3127) was dysregulated in multiple tumor types and has important roles in tumorigenesis and cancer progression. However, its biological roles and the mechanisms that regulate its expression in bladder cancer (BCA) remain to be determined. The expression level of miR-3127 was measured in BCA tissues and its cellular functions were examined using both *in vitro* and *in vivo* experiments. The interaction between miR-3127 and long non-coding RNA (lncRNA) LINC00319 was explored using RNA immunoprecipitation assay and luciferase reporter assays. We showed that miR-3127 expression was significantly downregulated in human BCA tissues and BCA cell lines. Lower miR-3127 levels were associated with worse survival in BCA patients. The overexpression of miR-3127 impaired BCA cell proliferation and invasion, and the knockdown of miR-3127 enhanced BCA cell proliferation and invasion *in vitro.* Importantly, miR-3127 was able to suppress cell growth *in vivo*. We demonstrated that miR-3127 repressed the proliferation and invasion of BCA cells though directly targeted the 3′-UTR of *RAP2A*, which served as a novel oncogene in BCA cells. The suppression of cell proliferation and invasion caused by miR-3127 overexpression could be partially abrogated by ectopic expression of RAP2A. Furthermore, high expression of LINC00319 was correlated with adverse survival in BCA patients. LINC00319 could bind directly with miR-3127 and inhibited its expression, and the tumor-promoting effects of LINC00319 could be reversed by re-expression of miR-3127 in BCA cells. Our findings indicated that lncRNA LINC00319-mediated miR-3127 repression promotes BCA progression through the upregulation of RAP2A. The re-introduction of miR-3127 or inhibition of LINC00319 might represent a promising therapeutic strategy for BCA treatment.

## Introduction

Bladder cancer (BCA) is the 9th most common cancer worldwide (Antoni et al., 2017). The American Cancer Society estimates that in 2018 there will be 81,190 new cases of BCA and 17,240 deaths in the United States (Siegel et al., 2018). During the past few years, the incidence and mortality rates of BCA have increased gradually in China (Pang et al., 2016). Approximately 15–30% of patients are diagnosed with muscle-invasive BCA, and many patients with muscle-invasive BCA develop a metastatic disease (Antoni et al., 2017). These patients with metastatic BCA often faced meager treatment options and a worse prognosis (Fletcher et al., 2011). Thus, a better understanding of the molecular basis of BCA is needed so that novel therapeutic approaches can be designed.

Both genetic and epigenetic alterations have been demonstrated to play important roles during bladder tumorigenesis and metastasis (Mitra et al., 2006). BCAs are generally characterized by alterations in the p53, PI3K/AKT and retinoblastoma pathways, and tumor angiogenesis further contributes to the neoplastic growth (Mitra et al., 2006). In particular, activation of the PI3K/AKT pathway appears to play a central role in the development of BCA (Mitra et al., 2006). RAP2A (a member of the small GTPase superfamily) mediates a variety of cellular processes such as proliferation, differentiation, cell adhesion, and cell cycle control (Bos, 2005). RAP2A has a key role in enhancing migration, invasion and metastasis by upregulating the phosphorylation level of AKT (Wu et al., 2015). RAP2A expression was dramatically increased in renal cell carcinoma tissues compared with normal renal tissues, and the ectopic expression of RAP2A enhanced the migration and invasive ability of cancer cells through an AKT-dependent mechanism (Wu et al., 2017). However, the expression and function of RAP2A has not been fully elucidated in the development of human BCA.

Epigenetic alterations, including DNA methylation, histone modification and non-coding RNAs play pivotal roles in the development of BCA (Porten, 2018). Long non-coding RNAs (lncRNAs) are a cluster of non-coding RNAs longer than 200 nucleotides, with little or no protein-coding potential (Xia et al., 2018). Many lncRNAs are reported to influence gene expression by working as guides, dynamic scaffolds and molecular decoys (Balas and Johnson, 2018; Dong et al., 2019). In human tumors, aberrant expression of lncRNAs is associated with tumorigenesis, metastasis and chemotherapy resistance (Balas and Johnson, 2018; Dong et al., 2019). In addition, microRNAs (miRNAs) are small endogenous non-coding RNAs composed of approximately 19–24 nucleotides that regulate target genes post-transcriptionally (Dong et al., 2017), and miRNAs play key roles in the modulation of tumor growth and metastasis (Dong et al., 2017, 2018b). It has been reported that miR-3127 acts as either a tumor suppressor or an oncogene in cancers, such as glioma (Pan et al., 2019), lung cancer (Sun et al., 2014; Yang et al., 2018) and hepatocellular carcinoma (Jiang et al., 2015). Downregulation of miR-3127-5p promotes epithelial-mesenchymal transition through activating the Wnt/FZD4/β-catenin pathway in non-small-cell lung cancer (Yang et al., 2018). However, miR-3127 promotes the proliferation and tumorigenesis in hepatocellular carcinoma by inducing the AKT/FOXO1 signaling (Jiang et al., 2015). Recent investigation has shown that several lncRNAs (including GACAT3, FOXD2-AS1, and LINC00319) modulate the malignant properties of tumor cells through sponging miR-3127 (Pan et al., 2019; Yang et al., 2020; Zhang and Chen, 2020). Thus, miR-3127 appears to be a central regulator connecting cancer progression with a wide range of cancer-related lncRNAs. Currently, the role of miR-3127 in BCA progression and the mechanisms that regulate miR-3127 expression were unclear.

In this study, we discovered that the expression of miR-3127 was reduced in BCA tissues and BCA cell lines. Enforced expression of miR-3127 impaired BCA cell proliferation and invasion, and repressed tumor growth *in vivo*. We have identified oncogene *RAP2A*, which was directly repressed by miR-3127 in BCA cells. Furthermore, we showed that lncRNA LINC00319 sponged miR-3127 and inhibited the levels of miR-3127, thereby promoting the proliferation and invasion of BCA cells. Lower levels of miR-3127 or higher expression of LINC00319 were correlated with adverse survival in BCA patients. Taken together, our results support the tumor-suppressive roles of miR-3127 in BCA cells, and gave a potential explanation for decreased miR-3127 expression in BCA.

## Materials and Methods

### Patients and Tissue Specimens

A total of 50 paired BCA tissue and adjacent normal tissues were included in this study. This study was approved by the Clinical Research Ethics Committee of Shangqiu First People’s Hospital of Henan, and written consent was obtained from each patient before sample collection. Fresh tissues were stored at −80°C before use.

### Cell Culture and Transfection

Human BCA cell lines (a grade 1 cancer cell line SW780 and a grade 3 cancer cell line T24) and human normal urothelial cell line SV-HUC-1 were obtained from Shanghai Institute of Cell Biology (Shanghai, China). T24 and SW780 cells were cultured in RPMI-1640 medium (Gibco, Waltham, MA, United States) supplemented with 10% fetal bovine serum (FBS). SV-HUC-1 cells were propagated in DMEM/F12 medium (Invitrogen, Carlsbad, CA, United States) supplemented with 10% FBS. We purchased the RAP2A or LINC00319 overexpression plasmids or the control plasmid from GenePharma (Shanghai, China). The siRNAs targeting LINC00319 or RAP2A, control siRNA, miR-3127 mimic, control mimic, miR-3127 inhibitor and control inhibitor were obtained from IGEbio (Guangzhou, China). Lipofectamine 3000 (Invitrogen, Waltham, MA, United States) was used according to the manufacturer’s protocol.

### RNA Extraction and Quantitative RT-PCR (qRT-PCR) Analysis

Total RNA was extracted from tissues or cells using TRIzol reagent (Invitrogen, Carlsbad, CA, United States), and cDNA was synthesized from 1 μg of RNA with an M-MLV Reverse Transcriptase Kit (Invitrogen, Carlsbad, CA, United States). The qRT-PCR analysis was performed using the SYBR Green quantitative real-time PCR Master Mix kit (Toyobo, Osaka, Japan) on an ABI-7500 RT-PCR system (Applied Biosystems). The primers for LINC00319, *RAP2A* and *GAPDH* were obtained from GenePharma (Shanghai, China). For the measurement of miR-3127 expression, we used the mirVanaTM qRT-PCR microRNA Detection Kit (Ambion, Austin, TX, United States) according to the manufacturer’s instructions. The relative expression of miR-3127 was normalized against that of the U6 endogenous control.

### Cell Proliferation Assay

Cell proliferation assay was performed using Cell Counting Kit-8 (CCK-8) assay (Dojindo, Japan) according to the manufacturer’s instructions. 5000 BCA cells were seeded into a 96-well plate and cultured with 100 μl of 10% FBS in the culture medium, and 10 μl of CCK-8 reagent was added into each well and incubated at the scheduled time points. The absorbance was measured at 450 nm by a microplate reader (Bio-Rad, Hercules, CA, United States). Each experiment was performed in triplicate.

### Matrigel Cell Invasion Assay

Transwell invasion assay was performed as described previously (Dong et al., 2018a). 2 × 10^4^ BCA cells in serum-free medium were seeded in the upper wells of Matrigel**-**coated Transwell plates (Corning Costar Co., Lowell, CA, United States). The medium containing 10% FBS was added to the lower chamber. After culturing for 24 h, the membranes were treated with 10% formaldehyde for 3 min, and stained with 2% crystal violet for 15 min at room temperature. Cells that invaded across the transwell membrane were counted using a light microscope in 10 randomly selected high-power fields.

### Western Blotting Analysis

Bladder cancer cells were lysed with cell lysis buffer (Beyotime, Guangzhou, China) supplemented with a protease inhibitor cocktail (Merck, Darmstadt, Germany). Protein concentrations of the total protein extracts were measured using a Bicinchoninic Acid Assay kit (Pierce, Rockford, IL, United States). 20 μg proteins were applied to 15% SDS-PAGE gel and transferred to a PVDF membrane (Millipore, Bedford, MA, United States). The membranes were then probed with primary antibody for RAP2A (1:2000, Santa Cruz, CA, United States) and GAPDH (1:5000, Santa Cruz, CA, United States) at 4°C overnight, incubated with horseradish peroxidase (HRP)-conjugated secondary antibody (in 5% fat-free milk) for 2 h, and finally visualized using the ECL reagent (Amersham Biosciences, Buckinghamshire, United Kingdom). GAPDH served as the loading control.

### Luciferase Reporter Assay

The luciferase reporter vectors containing wild-type LINC00319 (LINC00319-WT) and mutant LINC00319 (LINC00319-MUT), or wild-type *RAP2A* 3′-UTR (RAP2A-WT) and mutant *RAP2A* 3′-UTR (RAP2A-MUT), were constructed by GenePharma (Shanghai, China). BCA cells were co-transfected with 100 ng reporter plasmid containing LINC00319 (WT or MUT) or *RAP2A* 3′-UTR (WT or MUT) and 30 nM miR-3127 mimic or miR-3127 inhibitor using Lipofectamine 3000 reagent (Invitrogen, Waltham, MA, United States). Forty-eight hours later, the relative luciferase activity was measured with the Dual-Luciferase Reporter Assay System (Promega, Madison, WI, United States). Mutated LINC00319 or mutated *RAP2A* 3′-UTR was constructed by GenePharma (Shanghai, China) using the QuikChange Lightning Site-Directed Mutagenesis kit (Agilent Technologies, Santa Clara, CA, United States).

### RNA Immunoprecipitation Assay

RNA Immunoprecipitation (RIP) assays were performed to investigate whether LINC00319 could bind with miR-3127 using the Magna RIP RNA-Binding Protein Immunoprecipitation Kit (Millipore, Bedford, MA, United States) according to the manufacturer’s instructions. Briefly, cells were lysed in RIP lysis buffer, and the extracts were incubated with magnetic beads conjugated to human anti-Argonaute2 (Millipore, Bedford, MA, United States) or normal mouse IgG (Millipore, Bedford, MA, United States). The beads were incubated with Proteinase K to remove proteins. Finally, the purified RNAs were subjected to qRT-PCR analysis to detect the expression of LINC00319.

### Lentiviral Transfection

MiR-3127-overexpression lentiviral vector and control lentiviral vector, as well as miR-3127-sponge lentiviral vector and control lentiviral vector, were purchased from GenePharma (Shanghai, China). Lentivirus preparation and *in vitro* infection were performed as previously reported (Peng et al., 2019). In brief, T24 cells were infected by miR-3127-overexpression lentiviral vector or control vector, and SW780 cells were infected by miR-3127-sponge lentiviral vector or control vector. The stable cell lines were selected with 2 μg/ml puromycin (Sigma-Aldrich, Shanghai, China) for 14 days.

### Tumor Xenograft Experiments

The study was approved by the Institutional Animal Care and Use Committee of Shangqiu First People’s Hospital of Henan. BALB/c nude mice (4 weeks old) were purchased from Beijing HFK Bioscience (Beijing, China) and maintained under pathogen-free conditions. BCA cells (2 × 10^6^) with miR-3127 overexpression or inhibition were implanted subcutaneously into the right flank of the nude mice. Tumor growth was monitored using calipers and tumor volume measurement was performed every 3 days, using the following formula: volume = length (mm) × width^2^ (mm^2^)/2. After three weeks, the mice were sacrificed and the tumors were collected for immunohistochemical assay. The paraffin sections were refrigerated with anti-Ki-67 antibody the rabbit (1:1000, Abcam, Cambridge, United Kingdom) at 4°C overnight. After incubated with HRP-conjugated streptavidin, the sections were stained using 3, 3′-diaminobenzidine (ZLI9018, ZSGBBIO, China).

### Statistical Analysis

Statistical analysis was performed using SPSS 17.0 statistical software (SPSS, Chicago, United States). Statistical differences were determined using the Student’s *t*-test, one-way ANOVA test, or Wilcoxon signed-rank test. Values were expressed as the mean ± standard deviation of at least three independent experiments. *P-*values of less than 0.05 were considered statistically significant.

## Results

### MiR-3127 Expression Is Downregulated in Human BCA Tissues and BCA Cells

**FIGURE 1:**
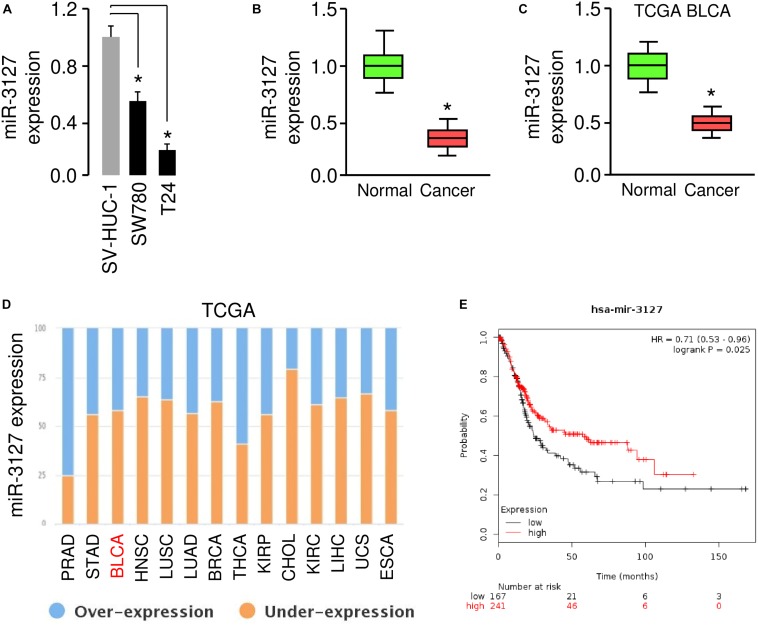
MiR-3127 is downregulated in human BCA **(A)**. Examination of miR-3127 expression in a normal urothelial epithelial cell line SV-HUC-1 and two BCA cell lines using qRT-PCR assays **(B)**. Expression of miR-3127 in human BCA tissues compared with matched adjacent normal tissues using qRT-PCR analysis **(C)**. Analysis of miR-3127 expression profiles in BCA samples and normal samples using the TCGA data retrieved in the MethHC database **(D)**. The expression trend for miR-3127 was analyzed in different cancer types using the BioXpress database **(E)**. The probability of overall survival in BCA patients expressing high or low miR-3127 levels assessed using the KMplotter database. *P < 0.05.

To investigate the role of the miR-3127 in BCA, we first used one normal urothelial epithelial cell line SV-HUC-1 and two BCA cell lines (SW780 and T24) to analyze miR-3127 expression in normal cells and BCA cells. The results of qRT-PCR analysis showed that miR-3127 levels were markedly decreased in BCA cells in comparison with SV-HUC-1 cells (Figure 1A). Consistent with the above findings, the expression of miR-3127 was downregulated in human BCA tissues compared with matched adjacent normal tissues, as examined by qRT-PCR assays (Figure 1B). In addition, we searched the MethHC database for miR-3127 expression in BCA tissues and normal tissues from the TCGA BCA datasets (Huang et al., 2015). We found that the levels of miR-3127 in BCA samples were significantly lower than those in the normal samples (Figure 1C). We also analyzed RNA-sequencing gene expression data of BCA samples and normal samples, which are available in the BioXpress database (Wan et al., 2015). The results suggested that miR-3127 expression was reduced in multiple cancer types including BCA (Figure 1D). To investigate the prognostic role of miR-3127 expression in BCA patients, we used an online database KMPlotter (Györffy et al., 2010). We used the median value to separate high and low groups for the survival analysis. The results showed that low expression of miR-3127 was significantly associated with poor overall survival in BCA patients (Figure 1E).

### MiR-3127 Inhibits the Proliferation and Invasion of BCA Cells

**FIGURE 2:**
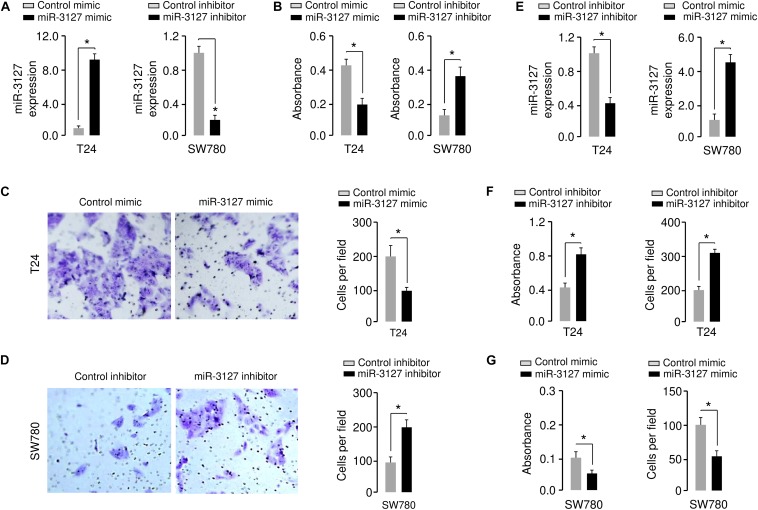
Overexpression of miR-3127 decreases cellular invasion and proliferation of BCA cells. **(A)** MiR-3127 levels in T24 cells transfected with miR-3127 mimic (or control mimic) and in SW780 cells transfected with miR-3127 inhibitor (or control inhibitor). **(B)** Proliferation assays in BCA cells after overexpression or knockdown of miR-3127. **(C,D)** Cell invasion assays in T24 cells transfected with miR-3127 mimic or control mimic **(C)**, and in SW780 cells transfected with miR-3127 inhibitor or control inhibitor **(D)**. **(E)** MiR-3127 levels in SW780 cells transfected with miR-3127 mimic (or control mimic) and in T24 cells transfected with miR-3127 inhibitor (or control inhibitor). **(F)** Proliferation and invasion assays in T24 cells after knockdown of miR-3127. **(G)** Proliferation and invasion assays in SW780 cells after overexpression of miR-3127. *P < 0.05.

Using qRT-PCR assays, we confirmed the upregulation of miR-3127 in T24 cells that were transfected with miR-3127 mimic, and the downregulation of miR-3127 in SW780 cells that were transfected with miR-3127 inhibitor (Figure 2A). We tried to explore the functions of miR-3127 in regulating the growth and invasiveness of BCA cells using CCK-8 assays and Matrigel invasion assays. The overexpression of miR-3127 significantly decreased the proliferation and invasion of T24 cells, while the downregulation of miR-3127 largely increased the abilities of SW780 cells to proliferate and invade (Figures 2B–D). To further validate these results, we transfected SW780 cells with miR-3127 mimic, and transfected T24 cells with miR-3127 inhibitor (Figure 2E). Then, we performed CCK-8 assays and Matrigel invasion assays and found that overexpression of miR-3127 in SW780 cells decreased cell proliferation and invasion, and knockdown of miR-3127 in T24 cells enhanced cell proliferation and invasion (Figures 2F,G). Taken together, our data supports an important role for miR-3127 in inhibiting the growth and invasiveness of BCA cells *in vitro*.

**FIGURE 3:**
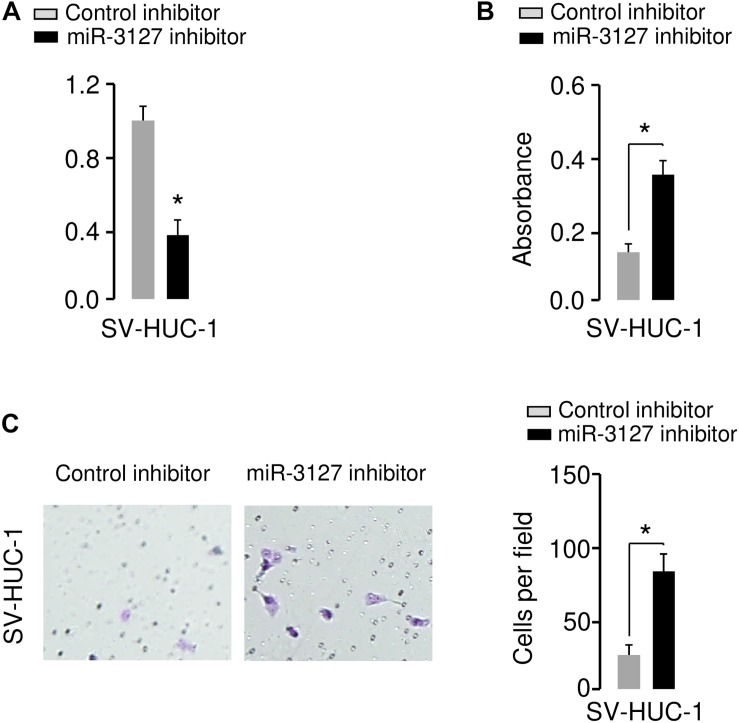
Downregulation of miR-3127 promotes cellular proliferation and invasion of SV-HUC-1 cells. **(A)** MiR-3127 expression in SV-HUC-1 cells transfected with miR-3127 inhibitor or control inhibitor. **(B,C)** Proliferation **(B)** and invasion **(C)** assays in SV-HUC-1 cells after knockdown of miR-3127. *P < 0.05.

We further investigated the functional role of miR-3127 in the human normal urothelial cell line SV-HUC-1. The inhibition of miR-3127 by using miR-3127 inhibitor dramatically decreased miR-3127 levels (Figure 3A), and significantly increased the growth and invasion of SV-HUC-1 cells (Figures 3B,C). These results indicate that downregulation of miR-3127 could increase cell proliferation and invasion of SV-HUC-1 cells.

### MiR-3127 Represses the Proliferation of BCA Cells *in vivo*

**FIGURE 4:**
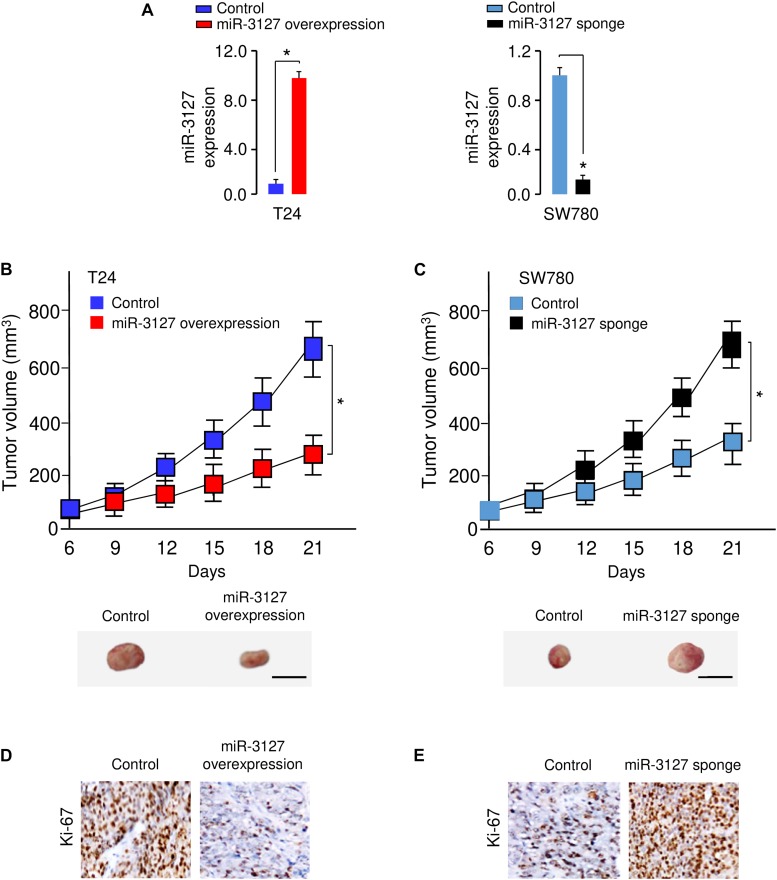
MiR-3127 overexpression impairs tumor growth *in vivo.* **(A)** MiR-3127-overexpression lentiviral vector or control lentiviral vector (control) was stably transfected into T24 cells, and miR-3127-silenced lentiviral vector or control lentiviral vector (control) was stably transfected into SW780 cells. The efficiency of miR-3127 overexpression and knockdown was verified using qRT-PCR analysis. **(B,C)** The stably transfected T24 **(B)** cells or SW780 **(C)** cells were subcutaneously injected into nude mice. The tumor growth curves were presented. Scale bar, 1 cm. **(D,E)** Immunohistochemical analysis of Ki-67 expression in tumors dissected from mice shown in panels **(B)** and **(C)**. *P < 0.05.

To examine whether miR-3127 could influence the growth of BCA cells *in vivo*, we transfected T24 cells with miR-3127-overexpression lentiviral vector or control lentiviral vector, and also transfected SW780 cells with miR-3127-silenced lentiviral vector or control lentiviral vector. The efficiency of stable miR-3127 overexpression or knockdown was verified by qRT-PCR analysis (Figure 4A). The stably transfected cells were then introduced into a nude mouse xenograft model, respectively. Three weeks post-injection of the BCA cells, the volume of tumors in mice injected with T24 cells transfected with miR-3127-overexpression lentiviral vector were significantly smaller than those in the control group (Figure 4B). Conversely, the lentivirus-mediated miR-3127 suppression significantly enhanced the proliferation of SW780 cells *in vivo* (Figure 4C). Immunohistochemistry-based studies revealed lower Ki-67 expression levels in the tumors from T24 cells overexpressing miR-3127 compared with those in the tumors from the control group, and higher Ki-67 expression in the tumors from SW780 cells transfected with miR-3127-silenced lentiviral vector (Figures 4D,E), indicating that miR-3127 reduces BCA cell growth *in vivo*.

### MiR-3127 Directly Targets and Suppresses the Expression of RAP2A

**FIGURE 5:**
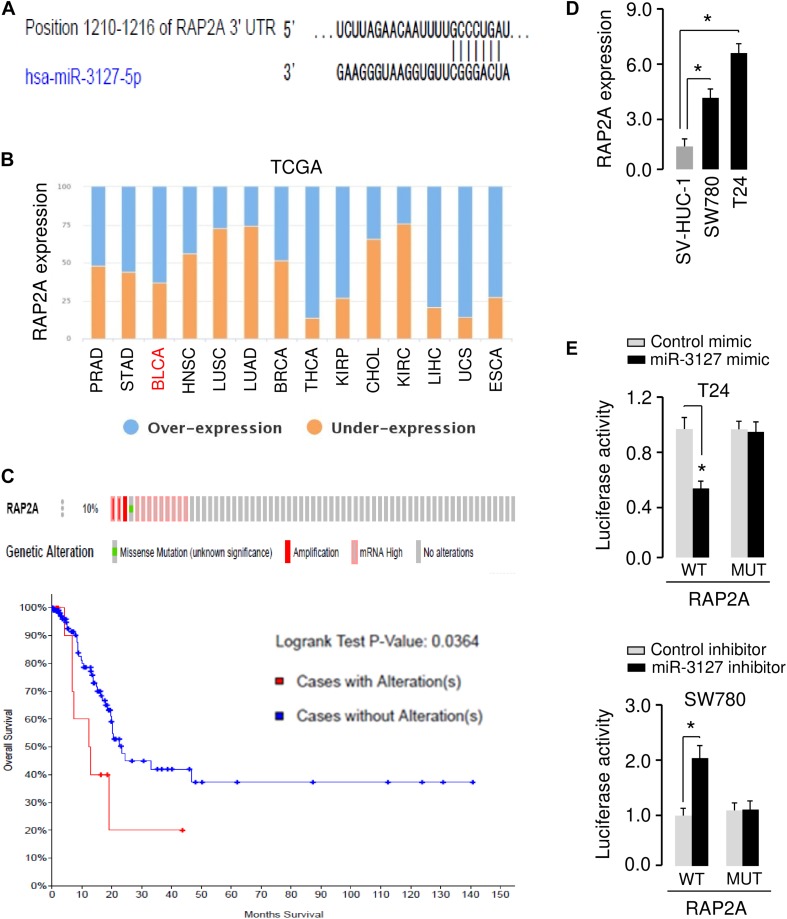
MiR-3127 directly targets and suppresses the expression of RAP2A. **(A)** Illustration of the predicted miR-3127 binding site in the *RAP2A* 3′-UTR. **(B)** The expression trend for RAP2A was analyzed in different cancer types using the BioXpress database. **(C)** Analysis of RAP2A alterations in BCA using the cBioPortal database (upper panel). Survival curves of patients without alterations in *RAP2A* (blue) compared to patients with genetic alterations in *RAP2A* (red) (bottom panel). **(D)** Examination of RAP2A expression in a normal urothelial epithelial cell line SV-HUC-1 and two BCA cell lines using qRT-PCR assays. **(E)** Luciferase reporter assays in BCA cells transfected with the wild type or mutant *RAP2A* 3′-UTR, along with miR-3127 mimic or miR-3127 inhibitor, respectively. *P < 0.05.

The possible target genes of miR-3127 were predicted using the target prediction database TargetScan. Among various candidates, *RAP2A* showed complementary sequences with miR-3127 (Figure 5A). RAP2A was believed to be a crucial upstream activator of the PI3K/AKT pathway in cancer cells (Wu et al., 2015, 2017). Thus, we analyzed the expression of RAP2A in tumor and normal tissue samples using the BioXpress database, and found that RAP2A was overexpressed in several tumor types including BCA (Figure 5B). The presence of mutation, copy number alteration and expression of RAP2A in BCA samples were recognized using the cBioPortal database (Gao et al., 2013). The alterations of RAP2A (including mutation, copy number amplification and mRNA overexpression) were found in 10% of all cases (Figure 5C). The alterations of RAP2A were significantly associated with worse overall survival of BCA patients (Figure 5C). Additionally, the expression level of RAP2A was found to be significantly higher in BCA cells when compared with normal cells (Figure 5D), suggesting a negative correlation between miR-3127 and RAP2A expression in BCA cells.

To validate the possible interaction between miR-3127 and RAP2A, we conducted luciferase reporter assays with WT *RAP2A* 3′-UTR or MUT *RAP2A* 3′-UTR reporter plasmids. We found that the overexpression of miR-3127 significantly reduced the luciferase activities of WT *RAP2A* 3′-UTR in T24 cells, and the downregulation of miR-3127 increased the luciferase activities of WT *RAP2A* 3′-UTR in SW780 cells (Figure 5E). The transfection with miR-3127 mimic or miR-3127 inhibitor in BCA cells had no significant effects on the luciferase activities of MUT *RAP2A* 3′-UTR (Figure 5E). These data suggest that RAP2A is a direct target of miR-3127 in BCA cells.

### RAP2A Overexpression Reverses the Inhibitory Effects of miR-3127 on BCA Cell Proliferation and Invasion

**FIGURE 6:**
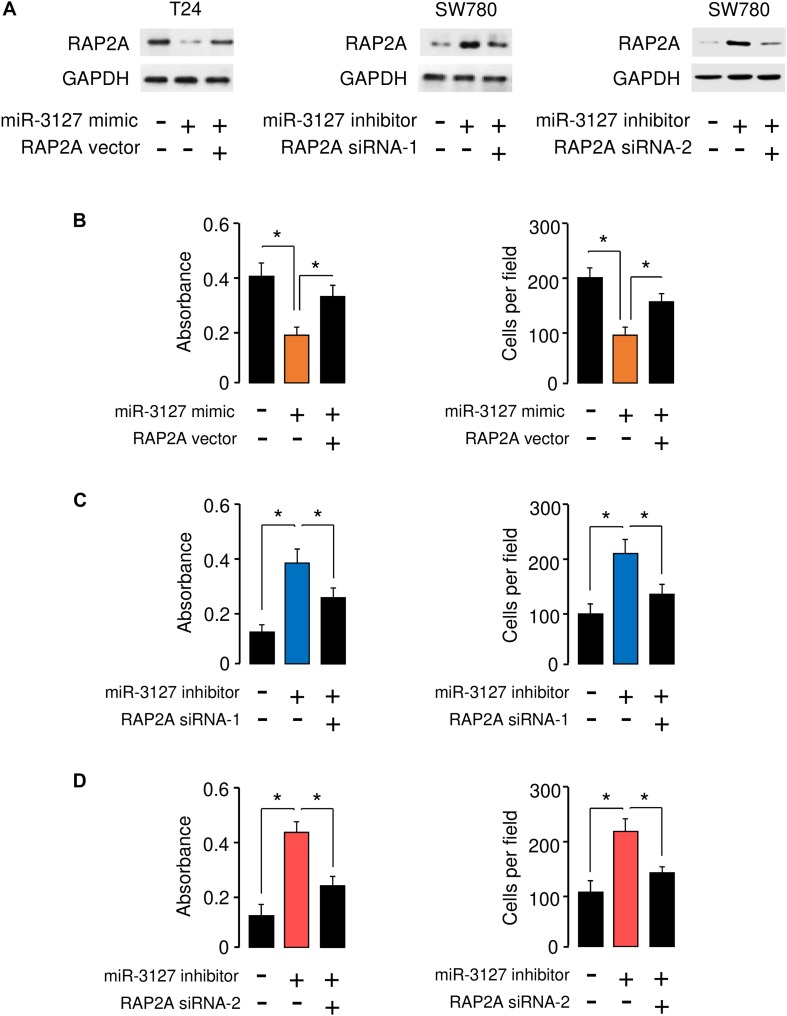
RAP2A overexpression reverses the inhibitory effects of miR-3127 on BCA cell proliferation and invasion. **(A)** The protein expression of RAP2A was examined in T24 cells transfected with control mimic or miR-3127 mimic, together with control vector or RAP2A vector (left panel), as well as in SW780 cells transfected with control inhibitor or miR-3127 inhibitor, together with control siRNA or RAP2A siRNA-1 (middle panel), or together with control siRNA or RAP2A siRNA-2 (right panel). **(B)** Proliferation and invasion assays in T24 cells transfected with control mimic or miR-3127 mimic, along with control vector or RAP2A expression vector. **(C,D)** Proliferation and invasion assays in SW780 cells transfected with control inhibitor or miR-3127 inhibitor, along with control siRNA or RAP2A siRNA-1 **(C)**, or along with control siRNA or siRNA 2 **(D)**. The minus sign indicated control mimic, control vector, control inhibitor, or control siRNA.*P < 0.05.

To determine whether miR-3127 repressed the proliferation and invasion of BCA cells by targeting RAP2A, we transfected RAP2A expression vector together with miR-3127 mimic into T24 cells, and transfected siRNAs that target RAP2A together with miR-3127 inhibitor into SW780 cells. Western blotting analysis suggested overexpression of miR-3127 decreased the protein expression of RAP2A in T24 cells transfected with miR-3127 mimic, while downregulation of miR-3127 increased the protein levels of RAP2A in SW780 cells (Figure 6A). The results form cell proliferation and invasion assays demonstrated that miR-3127 over-expression inhibited the proliferation and invasion of T24 cells, while the restoration of RAP2A expression significantly rescued the proliferation and invasion of T24 cells that were suppressed by miR-3127 overexpression (Figure 6B). We also found that the downregulation of miR-3127 elevated the proliferation and invasion of SW780 cells, and the knockdown of RAP2A significantly reversed these effects of miR-3127 silencing (Figures 6C,D). These results suggest that RAP2A is a functional target of miR-3127 in BCA cells.

### LINC00319 Sponges miR-3127 to Promote BCA Cell Proliferation and Invasion

**FIGURE 7:**
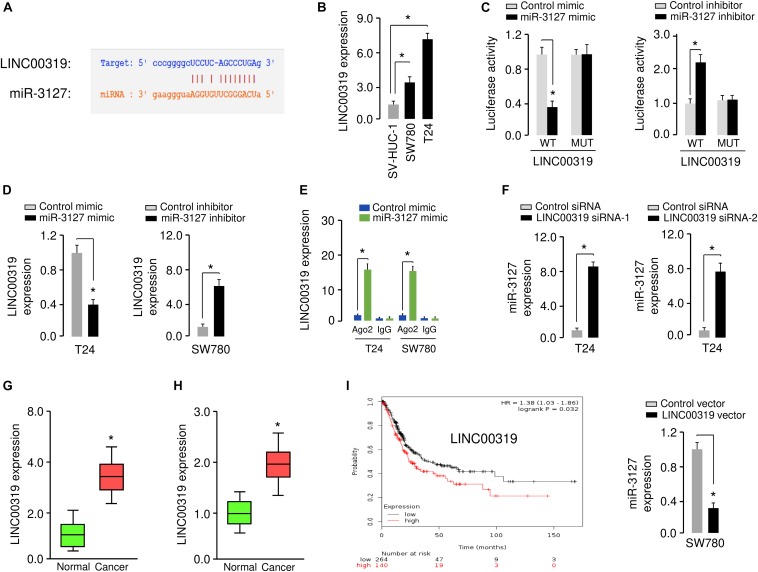
LINC00319 sponges miR-3127 to inhibit its levels in BCA cells. **(A)** The putative binding site for miR-3127 in the LINC00319 sequence. **(B)** Expression of LINC00319 in normal urothelial epithelial cells and BCA cell lines using qRT-PCR assays. **(C)** Luciferase reporter assays in BCA cells transfected with the wild type or mutant LINC00319, along with miR-3127 mimic or miR-3127 inhibitor, respectively. **(D)** Examination of LINC00319 expression in BCA cells following overexpression or knockdown of miR-3127. **(E)** RIP assays were done to verify the association between miR-3127 and LINC00319. **(F)** Expression of miR-3127 in BCA cells transfected with siRNAs targeting LINC00319 or LINC00319 expression vector. **(G)** Analysis of LINC00319 expression in human BCA tissues compared with matched adjacent normal tissues using qRT-PCR assays. **(H)** Analysis of LINC00319 expression in BCA samples and normal samples using the MethHC database. **(I)** The probability of overall survival in BCA patients expressing high or low LINC00319 levels assessed using the KMplotter database. *P < 0.05.

Increasing evidence has shown that lncRNAs could act as a sponge for miRNAs and inhibit their expression (Xiao et al., 2015). To test whether the expression of miR-3127 can be regulated by lncRNAs, we predicted the interactions between miR-3127 and lncRNAs using the starBase v2.0 database (Li et al., 2014), and observed the miR-3127-binding site in lncRNA LINC00319 (Figure 7A). Our qRT-PCR assays showed that LINC00319 levels were higher in BCA cells compared with normal cells (Figure 7B), indicating that the expression level of miR-3127 was negatively correlated with that of LINC00319. To further explore the association between miR-317 and LINC00319, the dual-luciferase reporter assay was performed. The transfection with miR-3127 mimics reduced the luciferase activity of WT LINC00319 reporter vector, but not that of MUT LINC00319 reporter vector (Figure 7C). On the other hand, the transfection with miR-3127 inhibitor induced the luciferase activity of the WT LINC00319 reporter vector, but did not affect the MUT LINC00319 reporter vector (Figure 7C). Our qRT-PCR results suggested that overexpression of miR-3127 reduced, whereas knockdown of miR-3127 increased the levels of LINC00319 in BCA cells (Figure 7D). Then, RIP assays were used to determine whether LINC00319 and miR-3127 exist in the same RNA-induced silencing complex. Compared with the control group transfected with control mimic, the endogenous LINC00319 was specifically enriched in the cells of miR-3127 mimic-transfected group (Figure 7E). Consistent with these data, qRT-PCR assays indicated that knockdown of LINC00319 could increase the expression of miR-3127 in T24 cells, and ectopic expression of LINC00319 in SW780 cells reduced the levels of miR-3127 (Figure 7F), indicating a reciprocal repression between miR-3127 and LINC00319 in BCA cells.

We analyzed the expression of LINC0319 in 50 paired BCA samples and normal samples using qRT-PCR assays. We found that LINC0039 was significantly upregulated in BCA tissues compared with adjacent normal tissues (Figure 7G). We also determined the levels of LINC00319 in BCA tissues and normal tissues using the TCGA data downloaded from the MethHC database. As expected, the expression of LINC00319 was increased in BCA samples compared with normal samples (Figure 7H). Finally, we assessed the association between LINC00319 expression and survival of BCA patients using the KMPlotter database. Patients with high LINC00319 expression levels had poorer overall survival (Figure 7I). Together, these results suggest that lncRNA LINC00319 may play an important role in the pathogenesis and prognosis of BCA.

### LINC00319 Promotes the Proliferation and Invasion of BCA Cells by Inhibiting miR-3127 Expression

**FIGURE 8:**
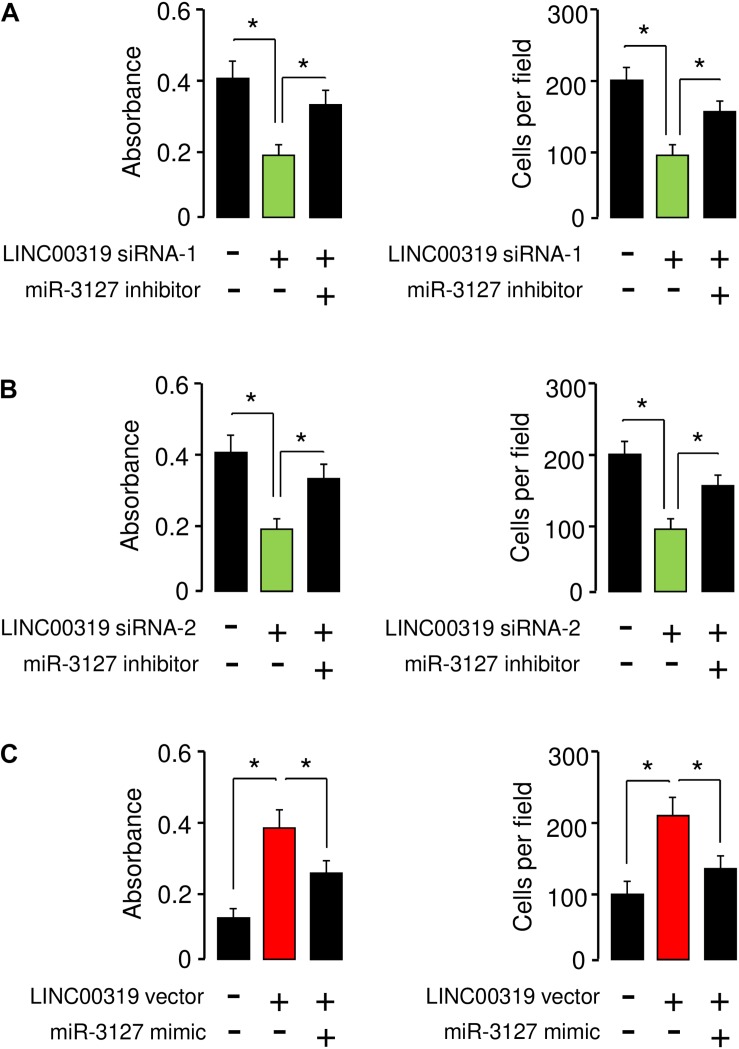
LINC00319 promotes the proliferation and invasion of BCA cells by inhibiting miR-3127 expression. **(A,B)** Cell proliferation and invasion assays in T24 cells transfected with control siRNA or LINC00319 siRNA-1, along with control inhibitor or miR-3127 inhibitor **(A)**, and in T24 cells transfected with control siRNA or LINC00319 siRNA-2, along with control inhibitor or miR-3127 inhibitor **(B)**. **(C)** Cell proliferation and invasion assays in SW780 cells transfected with control vector or LINC00319 expression vector, along with control mimic or miR-3127 mimic. The minus sign indicated control siRNA, control inhibitor, control vector, or control mimic. *P < 0.05.

We performed rescue assays to determine whether LINC00319 mediates the growth and invasion of BCA cells via inhibiting the expression of miR-3127. T24 cells were co-transfected with LINC00319 siRNAs and miR-3127 inhibitor, and SW780 cells were co-transfected with the LINC00319 expression vector and miR-3127 mimic. Our cell proliferation and invasion assays indicated that LINC00319 siRNAs-mediated suppression of cell proliferation and invasion was increased by the introduction of miR-3127 inhibitor in T24 cells (Figures 8A,B). In SW780 cells, overexpression of LINC00319 induced cell proliferation and invasion, which was suppressed by the transfection with miR-3127 mimic (Figure 8C). These data show that LINC00319 promotes BCA cell proliferation and invasion, at least in part through the downregulation of miR-3127.

## Discussion

There is a large unmet medical need for exploring the mechanism of BCA metastasis and developing new treatments for BCA patients with progressive disease (Bukhari et al., 2018). In this study, we identified the loss of miR-3127 expression in BCA samples using qRT-PCR assays and the TCGA RNA-sequencing data of human BCA. Concordantly, those patients harboring BCAs with lower levels of miR-3127 exhibited decreased overall survival. Then, our subsequent studies revealed that restoration of miR-3127 attenuated cell proliferation and invasion, partly through suppressing the expression of oncogene *RAP2A*. Further *in vivo* studies showed that overexpression of miR-3127 delayed tumor growth in a mouse model of BCA. Our results suggest that miR-3127 plays a tumor-suppressing role in BCA and thus could be a target for preventing BCA progression.

The functions of miRNAs depend on their target genes. For example, it has been reported that miR-3127 directly targets FZD4, thereby activating the Wnt/β-catenin signaling pathway in non-small-cell lung cancer (Yang et al., 2018). In another study, miR-3127 was shown to repress NSCLC cell proliferation and invasion via the direct regulation of oncogene *ABL1*, leading to the inhibition of the RAS/ERK pathway (Sun et al., 2014). However, there were still conflicting reports that miR-3127 actually acted as an oncogene in hepatocellular carcinoma by activating AKT/FOXO1 signaling, via directly targeting the 3′-UTR of *PHLPP1/2* (Jiang et al., 2015). The precise mechanisms of action and target genes of miR-3127 in BCA are not clear. RAP2A expression is increased in renal cell carcinoma tissues compared with normal renal tissues, and ectopic expression of RAP2A enhanced the migration and invasive ability of renal cell carcinoma cells at least by promoting the phosphorylation level of AKT (Wu et al., 2017). The results of the present study confirmed that RAP2A was a functional downstream effector of miR-3127 in BCA cells, and plays an important role in regulating the growth and invasiveness of BCA cells. Additional studies should be performed to determine the genes that were targeted by miR-3127.

Previous studies have shown that aberrant expression of lncRNAs affects the levels of miRNAs, thereby modulating the malignant phenotypes of tumor cells (Balas and Johnson, 2018; Dong et al., 2019). To date, the underlying mechanisms involved in the downregulation of miR-3127 in BCA remain unclear. Here, we have identified a novel lncRNA LINC00319 as a key regulator of miR-3127, and showed that LINC00319 can downregulate the expression of miR-3127 in BCA cells by functioning as a miRNA sponge. LINC00319 was highly expressed in many human tumors and associated with poorer survival of patients (Zhou et al., 2017; Li et al., 2018; Zhang et al., 2018, 2019; Song and Yin, 2019). LINC00319 was shown to promote the proliferative, migratory and invasive capabilities of tumor cells by stabilizing SIRT6 (Zhang et al., 2019) or by interacting with miR-1207 (Song and Yin, 2019), miR-450 (Zhang et al., 2018) and miR-32 (Zhou et al., 2017). Interestingly, a recent study revealed a direct interaction between lncRNA LINC00319 and miR-3127 in cervical cancer cells, and demonstrated that LINC00319 promotes migration, invasion and epithelial-mesenchymal transition process via regulating miR-3127 (Yang et al., 2020). Consistent with this finding, our data confirmed that LINC00319 directly bound to and reduced the expression of miR-3127 in BCA cells, indicating that LINC00319 and miR-3127 seem to be therapeutic targets in BCA treatment.

In summary, we found that LINC00319-mediated miR-3127 repression promotes BCA cell growth and invasion through upregulation of RAP2A. Therefore, enhancing the expression of miR-3127 or inhibition of LINC00319 expression might represent a promising therapeutic strategy for BCA treatment.

## Data Availability Statement

All datasets generated for this study has been included in the article.

## Ethics Statement

This study was approved by the Clinical Research Ethics Committee of Shangqiu First People’s Hospital of Henan. The patients/participants provided their written informed consent to participate in this study. The study was approved by the Institutional Animal Care and Use Committee of Shangqiu First People’s Hospital of Henan.

## Author Contributions

XW designed the experiments. All authors performed the experiments, analyzed the data, wrote the manuscript, and read and approved the final manuscript.

## Conflict of Interest

The authors declare that the research was conducted in the absence of any commercial or financial relationships that could be construed as a potential conflict of interest.
